# Evaluation study of single-anastomosis duodenal-ileal bypass with sleeve gastrectomy in the treatment of Chinese obese patients based on efficacy and nutrition

**DOI:** 10.1038/s41598-024-57289-3

**Published:** 2024-03-19

**Authors:** Lifu Hu, Lun Wang, Shixing Li, Yang Liu, Zheng Zhang, Minghao Xiao, Zhenhua Zhang, Zhiqiang Wei, Liang Cui, Tao Jiang

**Affiliations:** Department of Bariatric and Metabolic Surgery, China-Japan Union Hospital, Jilin University, Changchun, 130033 China

**Keywords:** Bariatric surgery, Obesity, Single-anastomotic duodenal-ileal bypass with sleeve gastrectomy, Type 2 diabetes mellitus, Nutrients, Endocrinology, Gastroenterology

## Abstract

To evaluate the efficacy and nutrition of single-anastomosis duodenal-ileal bypass with sleeve gastrectomy (SADI-S) in Chinese obese patients in the first postoperative year. Clinical data of 66 obese patients who underwent SADI-S surgery at China-Japan Union Hospital of Jilin University from November 2018 to May 2022 were retrospectively collected. The weight, body mass index (BMI), percentage of excess weight loss (%EWL), and percentage of total weight loss (%TWL) were recorded at 3, 6, and 12 months after surgery. Moreover, metabolic disease remission and nutrient deficiencies were assessed at 1 year postoperatively. Overall, 66 patients (38 males and 28 females) were recruited, with a mean age of 35 (18–61) years and an average preoperative BMI of 42.94 kg/m^2^. Before surgery, 38 patients had type 2 diabetes mellitus (T2DM), 46 patients had hyperuricemia (HUA), 45 patients had hypertension (HTN), 35 patients had hyperlipidemia, 12 patients had hypercholesterolemia, 12 patients had hyper-low-density lipoproteinemia, and 14 patients had gastroesophageal reflux disease symptoms (GERD). All patients had undergone a DaVinci robotic or laparoscopic SADI-S surgery, and none converted to laparotomy or died. Four patients developed postoperative complications and were cured and discharged after conservative treatment or surgical treatment. At 3, 6 and 12 months, the average %EWL was 62.07 ± 26.56, 85.93 ± 27.92, and 106.65 ± 29.65%, %TWL was 22.67 ± 4.94, 32.10 ± 5.18, and 40.56 ± 7.89%, respectively. Fasting blood glucose (FBG), glycated hemoglobin (HbA1c), uric acid (UA), triglycerides (TG), blood pressure (BP), and other indexes were significantly lower after one year post-surgery compared with the preoperative period (*P* < 0.05). The remission rates of T2DM, HUA, HTN, hypertriglyceridemia, hypercholesterolemia, and hyper-low-density lipoproteinemia 1 year after surgery were 100, 65.2, 62.2, 94.3, 100, and100%, respectively. One year after surgery, the remission rate of GERD was 71.4% (10/14), the rate of new occurrence of GERD was 12.1% (8/66), and the overall incidence rate was 18.2% (12/66). Except for vitamin B12(vit B12), the other nutrient indexes were significantly decreased after 1 year of surgery relative to levels before surgery (*P* < 0.05). The deficiency rates for vitamin A (vit A), vitamin E (vit E), zinc ion (Zn), and folic acid (FA) were higher (45.5, 25.8, 24.2, and 16.7%, respectively); however, there were no related clinical symptoms. SADI-S had significant effects on weight loss and metabolic disease remission. The main nutrient deficiencies after SADI-S were vit A, vit E, Zn, and FA deficiencies. The long-term efficacy and safety of SADI-S warrant further follow-up.

## Introduction

As of 2015, there were approximately 107.7 million obese children and 603.7 million obese adults worldwide. Approximately 4 million deaths worldwide are directly due to obesity, which accounts for 7.1% of all deaths^1^. Obesity can contribute to occurrence of many related metabolic diseases, such as T2DM, hyperlipidemia, and HTN including cardiovascular disease (CVD). Notably, obesity and related metabolic diseases have become serious public health problems in today’s society. Traditional treatment measures cannot effectively and permanently solve the problem of obesity and related metabolic diseases. Bariatric surgery, as an effective means of treating obesity and related metabolic diseases, has gradually attracted people’s attention and is widely accepted. SADI-S is one of the commonly performed bariatric procedures^2,3^. The first case of SADI-S was reported by Torres in 2007^4^. SADI-S is a simplified surgical procedure of duodenal switch (DS). It was initially designed to reduce the risk of malnutrition and other complications caused by DS^5^. Among the SADI-S variants with common channel lengths of 200 cm, 250 cm, and 300 cm, the SADI-S with a 300 cm common channel stands out with the most favorable outcomes. It not only leads to substantial weight loss but also diminishes the risk of malnutrition^5–7^. After nearly 16 years of experience accumulation, long-term follow-up results have shown that SADI-S has a very significant long-term effect on obesity and metabolic diseases^8,9^. However, the deficiencies of various nutrients and trace elements after SADI-S surgery are often ignored. Since October 2018, our center has used SADI-S as the main treatment method for severely obese patients, successively performing SADI-S in 160 cases so far. Therefore, this study aims to provide valuable guidance to bariatric metabolic surgeons and patients by retrospectively analyzing the clinical data of obese patients treated with SADI-S in our center and evaluating its efficacy, nutrient and trace element deficiencies.

## Materials and methods

### Patient information

A retrospective descriptive research method was adopted. The clinical data of 66 obese patients who underwent SADI-S from November 2018 to May 2022 were collected (53 cases with Da Vinci robot and 13 cases with traditional laparoscopy) in China-Japan Union Hospital of Jilin University (Changchun, China). Among them, 38 were males and 28 were females, with a mean age of 35 (18–61) years and an average preoperative BMI of 42.93 kg/m^2^. 38 patients had T2DM, 46 patients had HUA, 45 patients had HTN, 35 patients had hyperlipidemia, 12 patients had hypercholesterolemia, 12 patients had high low-density lipoproteinemia, and 14 patients had GERD. The study included a total of 23 women and 30 men in the robot-group (R-group), with an average age of 35.47 ± 7.72 years, preoperative weight of 125.82 ± 26.26 kg, and preoperative BMI of 42.37 ± 6.51 kg/m^2^. Among them, 29 individuals had type 2 diabetes before surgery, 33 had hyperuricemia, 33 had hypertension, 25 had hypertriglyceridemia, 11 had hypercholesterolemia, 11 had high low-density lipoproteinemia, and 12 had GERD. In the laparoscopic-group (L-group), there were 5 women and 8 men, with an average age of 34.38 ± 14.57 years, preoperative weight of 135.25 ± 25.62 kg, and preoperative BMI of 45.20 ± 7.82 kg/m^2^. Prior to the operation, 9 patients in this group had type 2 diabetes, 10 had hyperuricemia, 10 had hypertension, 10 had hypertriglyceridemia, 1 had hypercholesterolemia, 1 had high low-density lipoproteinemia, and 2 had GERD. Table 1 reports the Characteristics of the study population in the laparoscopic-group (L-group) and the robotic-group (R-group).

**Table 1:** Characteristics of the study population in the laparoscopic-group and the robotic-group.

Characteristic	R-group (n = 53)	L-group (n = 13)	Statistic 1	P-value
Age (years)	35.47 ± 7.72	34.38 ± 14.57	t = − 0.260	0.799
Gender (female/male)	23/30	5/8	*x*^2^ = 0.104	0.747
Preoperative BW^1^ (kg)	125.82 ± 26.26	135.25 ± 25.62	t = 1.166	0.248
Preoperative BMI^2^ (kg/m^2^)	42.37 ± 6.51	45.20 ± 7.82	t = 1.348	0.182
Preoperative related metabolic diseases				
T2DM^3^ (yes/no)	29/53	9/13	*x*^2^ = 10.974	< 0.001
HUA^4^ (yes/no)	36/53	10/13	*x*^2^ = 0.069	0.792
HTN^5^ (yes/no)	33/53	12/13	*x*^2^ = 0.745	0.388
Hyperlipidemia (yes/no)	25/53	10/13	*x*^2^ = 1.024	0.312
Hypercholesterolemia (yes/no)	11/53	1/13	*x*^2^ = 0.890	0.345
Hyper-low-density lipoproteinemia (yes/no)	11/53	1/13	*x*^2^ = 0.890	0.345
GERD^6^ (yes/no)	12/53	2/13	*x*^2^ = 0.222	0.638

^1^Body weight; ^2^Body mass index; ^3^Type 2 diabetes mellitus; ^4^Hyperuricemia; ^5^Hypertension; ^6^Gastroesophageal reflux disease symptoms.

All procedures were conducted in accordance with the Declaration of Helsinki. The study involving human participants were reviewed and approved by China-Japan Union Hospital of Jilin University (approval number: 2023102706). Informed consent was obtained from all individual participants included in the study.

### Inclusion and exclusion criteria

The inclusion criteria were: (I) For patients with severe obesity (BMI ≥ 40 kg/m^2^), we had recommended SADI-S, regardless of other related metabolic diseases; (II) For patients (27.5 kg/m^2^ < BMI < 40 kg/m^2^) with T2DM, we had recommended SADI-S, especially when the condition is severe. (ABCD score to assess preoperative diabetes severity). The exclusion criteria were: (I) revision surgery; (II) age < 16 or > 65 years old; (III) patients lost to follow-up and with incomplete data.

### Surgical methods

The upper duodenum was resected after a sleeve gastrectomy was done with a 34Fr gastric support tube. Then, an end-to-end anastomosis was performed between the proximal duodenum and the ileum, with the common channel retained at 300 cm.

### Observation indicators and evaluation criteria

#### Observation indicators

(I) *Perioperative conditions* operation time, postoperative hospital stay, and surgical complications; (II) *weight loss effect* BMI, %EWL and %TWL at 3, 6, and 12 months after the operation;(III) *remission rate of related metabolic diseases* FBG, HbA1c, BP, UA, TG, total cholesterol (TC), low-density lipoprotein (LDL), and various nutrients (trace elements, vitamins, FA, etc.) t 3, 6, and 12 months after the operation; (IV) The Clavien–Dindo classification was used to classify the severity of complications^10,11^.

#### Evaluation criteria

(I) EWL > 50% was the criterion for a successful weight loss surgery. (II) Metabolic disease remission was defined as follows: (a) T2DM: HbA1c < 6% without the use of hypoglycemic drugs; (b) hypertriglyceridemia: TG < 1.7 mmol/L; (c) hypercholesterolemia: TC < 5.7 mmol/L; (d) hyper-low-density lipoproteinemia: LDL < 4.13 mmol/L; (e) HTN: BP below 120/80 mmHg; (f) HUA: UA < 420 µmol/L; (g) GERD can be diagnosed if any of the following conditions are met: (i) have typical symptoms of GERD (heartburn, oesophageal chest pain and regurgitation), and experimental treatment with acid suppressants is effective; (ii) endoscopy shows reflux esophagitis grade B or above, reflux stenosis or Barrett’s esophagus (Pathologically confirmed); (iii) have atypical gastric reflux symptoms, no damage to the esophageal mucosa or reflux esophagitis grade A was found during endoscopy, and esophageal reflux monitoring suggests the presence of pathological reflux. GERD-Q score as an auxiliary diagnostic indicator^12^.

### Follow-up

All patients were followed up by professionals using outpatient service, telephone, or other methods, and relevant data were collected. Overall, 66 patients were followed up at 3, 6, and 12 months after operation. The follow-up indicators were BW, BMI, %EWL, %TWL, FBG, HbA1c, systolic blood pressure (SBP), diastolic blood pressure (DBP), UA, TG, TC, LDL, incidence of GERD and various nutrients (including trace elements, vitamins, FA, etc.) at 3, 6, and 12 months after surgery. The follow-up time ended in June 2023.

### Statistical analysis

SPSS 22.0 statistical software was used for data analysis. Normally distributed measurement data were expressed as mean ± standard deviation ($\overline{{\text{x}}}$ ± s) and compared using the *t*-test. Non-normally distributed measurement data were expressed as the median and interquartile range (IQR) and compared using the Wilcoxon rank sum test. Measurement data that are non-normally distributed and the two sets of variables are unordered were expressed as the yes/no and compared using the chi-square test. Statistical significance was set at a *P*-value of < 0.05.

### Ethical approval

All procedures were conducted in accordance with the Declaration of Helsinki. This study involving human participants were reviewed and approved by China-Japan Union Hospital of Jilin University (approval number: 2023102706).

### Informed consent

Informed consent was obtained from all individual participants included in the study.

## Results

### Perioperative conditions and the incidence of GERD

All 66 obese patients underwent SADI-S, with clear intraoperative vision and with no obvious bleeding, conversion to laparotomy, and death. The mean operation time was 194.79 ± 45.04 min. There were 4 cases of surgical complications, with an incidence rate of 6.1% (4/66), and surgical complications occurred in the first 33 cases. Among them, 3 patients were Clavien–Dindo grade II, with abdominal effusion, internal jugular vein thrombosis, and anastomotic leakage; 1 patient was Clavien–Dindo grade III, with gastric fistula. The patient with anastomotic leakage was cured and discharged from the hospital after symptomatic treatment such as fasting, water rehydration, and acid suppression. Patients with internal jugular vein thrombosis and peritoneal effusion were discharged after symptomatic and supportive treatment. The patient with gastric fistula was cured and discharged after surgical treatment. One year after surgery, among 14 patients with GERD, 10 patients had improved reflux symptoms and no obvious abnormality was found in endoscopy, and the remission rate of 71.4% (10/14). Moreover, 12 patients were complicated with GERD 1 year after surgery (8 of them were new-onset GERD and 4 were complicated with preoperative GERD), and the incidence rate was 18.2% (12/66). 8 patients diagnosed with new GERD all presented typical symptoms of GERD and had a GERD-Q score > 8, endoscopy revealed reflux esophagitis grade B or higher. Among the patients whose GERD was not relieved, endoscopy in 1 patient showed that the reflux esophagitis was worse than before surgery (Grade C vs Grade B).

### Treatment effect

#### Weight loss effect

The BMI of all patients continued to decrease significantly at 3, 6, and 12 months after operation (*P* < 0.05). None of the patients was underweight. The average %TWL and %EWL at 3, 6, and 12 months after operation was 22.67 ± 4.94, 32.10 ± 5.18, and 40.56 ± 7.89% and 62.07 ± 26.56, 85.93 ± 27.92, 106.65 ± 29.65%, respectively. Moreover, %EWL and %TWL increased significantly at 6 and 12 months after operation compared with those at 3 months after operation (Table 2).

**Table 2:** Changes in BW, BMI, TWL, and EWL before and after surgery.

Characteristic	Preoperative	3 months after surgery	6 months after surgery	12 months after surgery
BW^1^ (kg)	127.67 ± 26.21	98.58 ± 20.83	86.10 ± 16.42	74.64 ± 12.71
BMI^2^ (kg/m^2^)	42.93 ± 6.82	33.19 ± 5.74	29.03 ± 4.52	25.30 ± 3.66
%TWL^3^	–	22.67 ± 4.94	32.10 ± 5.18	40.56 ± 7.89
%EWL^4^	–	62.07 ± 26.56	85.93 ± 27.92	106.65 ± 29.65

	*P*-value^a^	*P*-value^b^	*P*-value^c^	
BW_1_ (kg)	0.000	0.000	0.000	
BMI_2_ (kg/m^2^)	0.000	0.000	0.000	
%TWL_3_	–	0.000	0.000	
%EWL_4_	–	0.000	0.000	

^1^Body weight; ^2^Body mass index; ^3^Percentage of total weight loss; ^4^Percentage of excess weight loss.

^a^Represents the comparison between preoperative and 3 months after operation.

^b^Represents the comparison between3 and 6 months after operation.

^c^Represents the comparison between6 and 12 months after operation.

#### Hypoglycemic effect

Among the 38 patients with T2DM, Preoperatively, the duration of T2DM was 2.7 (0–15) years as well as the ABCD score, which has been widely utilized to predict the postoperative efficacy of T2D patients^13^, was 8 (3–10). Further, before surgery, 2 received insulin therapy, 5 patients received oral hypoglycemic drugs, 9 patients received insulin combined with oral hypoglycemic drugs, and the remaining patients never took drugs or insulin therapy. Preoperative FBG and HbA1c were 8.48 (7.42, 10.28) mmol/L and 8.12 ± 1.69% respectively. FBG and HbA1c were 4.96 (4.61, 5.42) mmol/L and 4.97 ± 0.54% after one year of operation, respectively. The results showed that FBG and HbA1c were significantly reduced 1 year after operation, and the difference was statistically significant (*P* < 0.05). The complete remission rate of T2DM was 100% (38/38) (Table 3).

**Table 3:** Changes in metabolic disease indexes before operation and after 1 year of operation.

Characteristic	Preoperative	3-month after surgery	6-month after surgery	12-month after surgery		
FBG^1^ (mmol/L)	8.48 (7.42, 10.28)	5.30 (5.11, 5.81)	5.04 (4.71, 5.51)	4.96 (4.61, 5.42)		
HbA1c^2^ (%)	8.12 ± 1.69	5.53 ± 0.69	4.94 ± 0.41	4.97 ± 0.54		
UA^3^ (μmol/L)	540.86 ± 98.71	440.88 ± 91.88	390.70 ± 73.60	389.69 ± 94.31		
SBP^4^ (mmhg)	156.27 ± 18.49	133.69 ± 14.32	124.89 ± 11.90	122.69 ± 15.75		
DBP^5^ (mmhg)	92.18 ± 15.08	81.73 ± 12.45	77.96 ± 10.86	74.67 ± 12.53		
TG^6^ (mmol/L)	2.71 (2.30, 3.46)	1.70 (1.48, 2.16)	1.54 (1.09, 1.8)	1.03 (0.72, 1.27)		
TC^7^ (mmol/L)	7.07 ± 0.55	5.06 ± 0.85	4.48 ± 0.81	4.07 ± 0.67		
LDL^8^ (mmol/L)	4.57 ± 0.43	3.45 ± 0.71	2.92 ± 0.84	2.28 ± 0.63		

Characteristic	*P-*value^a^	*P*-value^b^	*P*-value^c^	Statistics 1	Statistics 2	Statistics 3
FBG^1^ (mmol/L)	< 0.001	< 0.001	< 0.001	Z = − 6.322	Z = − 6.940	Z = − 6.940
HbA1c^2^ (%)	< 0.001	< 0.001	< 0.001	t = 9.326	t = 11.537	t = 11.223
UA^3^ (μmol/L)	< 0.001	< 0.001	< 0.001	t = 5.922	t = 10.676	t = 9.858
SBP^4^ (mmhg)	< 0.001	< 0.001	< 0.001	t = 9.180	t = 11.459	t = 11.390
DBP^5^ (mmhg)	< 0.001	< 0.001	< 0.001	t = 4.938	t = 6.510	t = 7.324
TG^6^ (mmol/L)	< 0.001	< 0.001	< 0.001	Z = − 5.768	Z = − 6.379	Z = − 6.878
TC^7^ (mmol/L)	< 0.001	< 0.001	< 0.001	t = 9.465	t = 12.579	t = 11.574
LDL^8^ (mmol/L)	< 0.001	< 0.001	< 0.001	t = 6.779	t = 8.066	t = 11.402

Statistics 1: represents the comparison between preoperative and 3 months after operation.

Statistics 2: represents the comparison between preoperative and 6 months after operation.

Statistics 3: represents the comparison between preoperative and 1 year after operation.

^1^Fasting blood glucose; ^2^Glycated hemoglobin; ^3^Uric acid; ^4^Systolic blood pressure; ^5^Diastolic blood pressure; ^6^Triglycerides; ^7^Total cholesterol; ^8^Low-density lipoprotein.

^a^Represents the comparison between preoperative and 3 months after operation.

^b^Represents the comparison between preoperative and 6 months after operation.

^c^Represents the comparison between preoperative and 1 year after operation.

### Treatment effects of other related metabolic diseases

UA, SBP, DBP, TG, TC, LDL, and other indicators were significantly lower after one year of operation than those before operation (*P* < 0.05) (Table 3). The remission rates of HUA, HTN, hypertriglyceridemia, hypercholesterolemia, and hyper-low-density lipoproteinemia were 65.2% (30/46), 62.2% (28/45), 94.3% (33/35), 100% (12/12), and 100% (12/12), respectively (Table 3).

### Changes in various nutrient indicators

The average hemoglobin (HGB) at 3 months, 6 months, and 12 months after surgery were (143.91 ± 14.36) g/L, (138.19 ± 13.08) g/L, and (133.94 ± 18.55) g/L, respectively. Similarly, the average hematocrit (MCV) at 3 months, 6 months, and 12 months after surgery were 0.43 ± 0.05, 0.42 ± 0.04, and 0.41 ± 0.05, respectively, and the incidence of anemia was 6.1% (4/66). The symptoms of anemia in all 4 patients improved after a blood transfusion. The incidence of hypoalbuminemia and serum ferritin (SF) deficiency was 4.5% (3/66) and 4.5% (3/66), respectively. The protein levels of the 3 patients returned to the normal range after conservative treatment. All patients were regularly supplemented with various vitamins and calcium (Ca) after the operation. The incidences of vit A, vit E, FA, potassium(K), Ca, magnesium (Mg), iron (Fe) and Zn deficiencies were 45.5 (30/66), 25.8 (17/66), 16.7 (11/66), 3.0 (2/66), 4.5 (3/66), 3.0 (2/66), 13.6 (9/66), and 24.2% (16/66), respectively. No patient suffered from vit B12, vitamin D (vit D), sodium (Na) ion, chloride (Cl) ion, and phosphorus (P) ion deficiencies (Table 4).

**Table 4:** Changes in various nutrient indexes before operation and after 1 year of operation.

characteristic	Normal range	Preoperative	3 months after surgery	6 months after surgery	1 year after surgery	
HGB^1^	120-160g/L	150.24 ± 18.20	143.91 ± 14.36	138.19 ± 13.08	133.94 ± 18.55	
MCV^2^	0.37–0.48	0.45 ± 0.05	0.43 ± 0.05	0.42 ± 0.04	0.41 ± 0.05	
TP^3^	62–83 g/L	72.59 ± 5.01	71.22 ± 4.31	69.49 ± 3.86	68.32 ± 5.54	
ALB^4^	35–52 g/L	42.47 ± 3.17	41.81 ± 3.32	40.96 ± 3.35	41.41 ± 3.86	
SF^5^	12–135 ng/L	147.79 ± 123.60	196.66 ± 171.59	165.82 ± 160.25	162.53 ± 154.20	
FA^6^	> 3.2 ng/mL	13.02 ± 5.89	10.60 ± 5.75	8.95 ± 6.47	8.60 ± 5.98	
VitA^7^	0.38–0.98 ug/mL	0.55 ± 0.16	0.40 ± 0.15	0.38 ± 0.11	0.41 ± 0.14	
VitD^8^	3–29 ng/mL	13.77 ± 6.58	12.46 ± 6.07	12.52 ± 7.21	13.18 ± 8.02	
VitE^9^	5.7–19.9 ug/mL	10.27 ± 3.28	8.20 ± 2.61	7.93 ± 2.23	7.67 ± 2.64	
vitB12^10^	180–916 pg/mL	395.05 ± 184.79	583.88 ± 274.17	586.34 ± 288.87	622.43 ± 323.28	
Ca^11^	2.2–2.65 mmol/L	2.38 ± 0.12	2.41 ± 0.12	2.39 ± 0.15	2.35 ± 0.15	
Mg^12^	0.8–1.0 mmol/L	0.84 ± 0.06	0.89 ± 0.05	0.89 ± 0.06	0.90 ± 0.09	
Fe^13^	8.9–32.3 mmol/L	14.73 (10.69, 17.91)	12.86 (10.84, 15.41)	13.25 (9.52, 17.73)	14.04 (11.55, 19.27)	
Zn^14^	10.7–19.5 mmol/L	14.63 (13.25, 17.73)	15.53 (14.00, 18.56)	13.25 (11.96, 14.66)	11.65 (10.35, 12.80)	
K^15^	3.5–5.2 mmol/L	4.13 ± 0.31	4.06 ± 0.37	4.08 ± 0.37	4.14 ± 0.31	
Cl^16^	96–108 mmol/L	103.52 ± 2.73	104.90 ± 2.87	105.06 ± 2.07	106.21 ± 2.37	
Na^17^	136–145 mmol/L	139.43 ± 2.51	141.39 ± 2.19	141.27 ± 1.45	141.25 ± 1.77	
P^18^	0.81–1.45 mmol/L	1.25 ± 0.18	1.17 ± 0.18	1.29 ± 0.19	1.31 ± 0.18	

Characteristic	*P*-value^a^	*P*-value^b^	*P*-value^c^	Statistics 1	Statistics 2	Statistics 3
HGB^1^	0.001	< 0.001	< 0.001	t = 3.464	t = 6.890	t = 7.382
MCV^2^	0.002	< 0.001	< 0.001	t = 3.190	t = 5.659	t = 7.252
TP^3^	0.088	< 0.001	< 0.001	t = 1.731	t = 4.075	t = 4.779
ALB^4^	0.171	0.007	0.063	t = 1.386	t = 2.771	t = 1.895
SF^5^	0.009	0.317	0.430	t = − 2.688	t = 1.008	t = − 0.794
FA^6^	< 0.001	< 0.001	< 0.001	t = 3.864	t = 5.033	t = 5.252
VitA^7^	< 0.001	< 0.001	< 0.001	t = 6.911	t = 8.053	t = 6.559
VitD^8^	0.098	0.128	0.476	t = 1.680	t = 1.540	t = 0.718
VitE^9^	< 0.001	< 0.001	< 0.001	t = 6.293	t = 5.950	t = 5.986
vitB12^10^	< 0.001	< 0.001	< 0.001	t = − 6.073	t = − 5.885	t = − 6.356
Ca^11^	0.066	0.432	0.225	t = − 1.869	t = − 0.790	t = 1.226
Mg^12^	< 0.001	< 0.001	< 0.001	t = − 6.042	t = − 5.410	t = − 4.651
Fe^13^	0.062	0.264	0.973	Z = − 1.866	Z = − 1.117	Z = − 0.034
Zn^14^	0.046	< 0.001	< 0.001	Z = − 1.996	Z = − 3.664	Z = − 6.722
K^15^	0.208	0.415	0.829	t = 1.272	t = 0.820	t = − 0.217
Cl^16^	< 0.001	< 0.001	< 0.001	t = − 4.326	t = − 3.807	t = − 6.974
Na^17^	< 0.001	< 0.001	< 0.001	t = − 6.027	t = − 5.413	t = − 6.264
P^18^	0.002	0.196	0.013	t = 3.254	t = − 1.307	t = − 2.541

Statistics 1: represents the comparison between preoperative and 3 months after operation.

Statistics 2: represents the comparison between preoperative and 6 months after operation.

Statistics 3: represents the comparison between preoperative and 1 year after operation.

^1^Hemoglobin; ^2^Hematocrit; ^3^Total protein; ^4^Albumin; ^5^Serum ferritin; ^6^Folic acid; ^7^Vitamin A; ^8^Vitamin D; ^9^Vitamin E; ^10^Vitamin B12; ^11^Calcium; ^12^Magnesium; ^13^Iron; ^14^Zinc; ^15^Potassium; ^16^Chloride; ^17^Sodium; ^18^Phosphorus.

^a^Represents the comparison between preoperative and 3 months after operation.

^b^Represents the comparison between preoperative and 6 months after operation.

^c^Represents the comparison between preoperative and 1 year after operation.

## Discussion

This study presents the findings of a single-center evaluation of the efficacy of SADI-S, along with an assessment of various nutrient and trace element deficiencies. Notably, this study boasts the largest sample size in China. The results demonstrate that SADI-S effectively promotes weight loss and provides relief from metabolic diseases. Moreover, the incidence of postoperative surgical and nutritional complications was relatively low, indicating that SADI-S is a safe and viable procedure. It was found that the average %EWL was 62.1, 85.9, and 107% at 3, 6, and 12 months after surgery, respectively, which were all far greater than the recommended threshold of 50%^14^ and the mean %EWL of 80 and 100% at 6 and 18 months after surgery, respectively, reported by Sanchez-Pernaute et al. Several studies^3,15^ have compared the weight loss efficacy between SADI-S and SG, consistently showing that SADI-S yields superior results. Data from our center^16^ further support this, demonstrating a significantly better weight loss effect with SADI-S compared to SG 12 months post-surgery (total weight loss rate: 38.5% vs 29.5%, P < 0.001). Gomberawalla et al.^17^ highlighted that higher BMI indexes are associated with poorer weight loss outcomes following SG. A review on weight regain post-SG surgery revealed a wide range of weight regain rates, from 5.7 to 75.6%^18^. Conversely, some studies have indicated that SADI-S leads to no weight regain within 2 years post-surgery^19^. The weight loss effect was much higher than that of sleeve gastrectomy (SG), Roux-en-Y gastric bypass (RYGB), etc.^20–22^, which may be because SADI-S has a different weight loss mechanism than other surgeries. SADI-S not only restricts food intake through gastric sleeve resection, but also performs duodenal ileal anastomosis while leaving a longer small intestine to reduce absorption.

Our data also showed that the complete remission rate of T2DM at 12 months after SADI-S was 100%, which is much higher than the 60–80% reported in previous studies^7,23,24^. Possible reasons for the difference in efficacy are as follows: (1) a smaller gastric tube may result in better efficacy, as we used a 34Fr gastric tube compared with a (36Fr–42Fr) tube used by previous studies. (2) The type 2 diabetes patients in this study had a shorter duration and milder disease. This mean that a longer duration of type 2 diabetes and the combined use of hypoglycemic drugs may lead to more severe damage to pancreatic islet function, ultimately extending the remission period post bariatric surgery. Some studies^25–27^ have pointed out that as the preoperative ABCD score decreases, the type 2 diabetes remission rate of OAGB, RYGB, and SG also decreases, while SADI-S can still maintain a higher type 2 diabetes remission rate. The complete remission rate of T2DM in the current study was similar to that of Surve et al. (94.4%)^28^. Enoch et al.^3^ conducted a comparative study on the efficacy of SADI-S, RYGB, and SG in the treatment of diabetes. Their findings indicated that SADI-S was significantly more effective than RYGB and SG one year after surgery. This was corroborated by Cottam et al.^29^. Additionally, our previous research^16^ demonstrated that the complete remission rate of type 2 diabetes one year after SADI-S surgery was higher compared to the SG group (100% vs 75%). SADI-S can also maintain good efficacy in treating patients with severe diabetes^30^, and is better than SG and gastric bypass^25^. Lee et al.^31^ also highlighted that a total weight loss percentage (TWL%) of less than 20% post-SG surgery is associated with a poor prognosis for diabetes. Based on the results of this study and previous references^26,30,32^, it can be inferred that SADI-S has great advantages in the treatment of obesity in patients with T2DM, which may be because SADI-S almost covers the remission of all T2DM, with potential mechanisms including appropriate gastric volume reduction, reduced caloric intake, reduced intestinal absorption, etc.^33,34^. The same argument may also explain the remission of hyperlipidemia^35^. Similar to the complete remission rate of T2DM in our study, 94.3% of patients had complete remission of hypertriglyceridemia, 100% of patients had complete remission of hypercholesterolemia, 100% of patients had complete remission of hyper-low-density lipoproteinemia, while 62.2% of patients had remission of HTN. GERD after SADI-S surgery has always been a controversial topic, because GERD is not only a disease complicated by obesity, but also one of the postoperative complications of SADI-S. In this study, one year after surgery, the incidence rate of GERD was 12.1% (8/66). One possible explanation for this phenomenon could be attributed to the distinctive ring structure of SADI-S, which may introduce an additional risk of bile reflux and be linked to carcinogenesis^27^. Notably, bile reflux is a known contributor to GERD. As emphasized by Yashkov et al., both the distance of the duodenal transection from the pylorus and the initial pyloric insufficiency might potentially be associated with bile reflux^28^. Consequently, determining the optimal adjustment for intestinal anastomosis and the distance between gastric antrum resection and pylorus may serve as a crucial strategy for addressing GERD following SADI-S surgery. This necessitates further research and investigation to validate and explore.

Substantial weight loss in a short period is always accompanied by changes in some nutrients in the body, which reflects the metabolic transformation process of the body. Hence, a single nutrient index cannot fully evaluate the nutritional status of patients after surgery. Samples were collected and analyzed for levels of HGB, total protein (TP), albumin (ALB), FA, and various vitamins, which were used to evaluate the safety of SADI-S in Chinese obese patients^36^. We observed anemia in 6% of the patients who were all young women of childbearing age; however, the HGB of these patients was slightly lower than the lower limit of the normal value. The prevalence of anemia after biliopancreatic diversion (BPD) is estimated to be about 40%, which can be reduced to 5% by appropriate Fe and FA supplementation^37^. Our study found that the symptoms of anemia improved after iron and FA supplementation. Of the 66 patients, 3 (4.5%) developed hypoalbuminemia, and none of them followed the high-protein diet recommended by the nutritionist after surgery. By conservative treatment, the ALB levels of the 3 patients were restored to normal. Sanchez-Pernaute et al.^38^ found that 34 and 13.7% of patients had abnormal TP and ALB levels after 1 year, respectively. The discrepancy in results may be because Sanchez-Pernaute et al. used SADI-S with a 200–250 cm common channel, which is much shorter than the 300 cm used in our study. While using the same common channel of 300 cm, Surve et al.^28^ found that the incidence of hypoalbuminemia was 6.2% after 1 year of SADI-S, which is consistent with our result. Furthermore, we found that vit A and vit E deficiencies were the most common nutrient deficiencies, with an incidence rate of 45.5 and 25.8%, respectively. Interestingly, Rao et al.^39^ reported that the nutrient deficiency rate was 0% after 4 years of SADI-S surgery. This may be because the postoperative follow-up rate was high and nutrients were monitored and supplemented in time. The lack of vitamins and various trace elements after surgery may be because most patients in our study were not followed up at 3 and 6 months after surgery and therefore various nutrients were not monitored and supplemented in time. SADI-S as an emerging bariatric technique still needs to be explored, as Shoar et al. previously pointed out, there is a trend to establish a longer common pathway to reduce nutrient deficiencies while maintaining glycemic control and weight loss in a balanced state^24^.

Importantly, approximately 80% (53/66) of the patients underwent SADI-S through the da Vinci robotic surgical system in the present study. This surgical system is increasingly used in bariatric surgery. The application of the da Vinci robotic system in patients with a BMI of > 50 kg/m^2^ or a history of abdominal surgery has been proven to help alleviate the doctor’s torque fatigue for patients with particularly thick abdominal walls^40^. Other studies have also shown that the use of the da Vinci robotic surgery system for bariatric surgery in patients with a BMI of > 50 or > 60 kg/m^2^ can reduce the incidence of postoperative complications and shorten the length of hospital stay^41^. Our study showed that only one patient with da Vinci robot SADI-S developed a gastric fistula after surgery, which occurred before the operator passed the learning curve of da Vinci robot SADI-S^42^, and after passing the learning curve, no surgical complications occurred in the patient.

## Conclusion

In summary, SADI-S has a significant short-term effect on obesity and related metabolic diseases in China, and the postoperative nutritional status and complications are within an acceptable range. However, its long-term efficacy and safety still need further follow-up observation.

## Data Availability

The datasets used and/or analyzed during the current study available from the corresponding author on reason-able request.

## References

[1] GBD 2015 Obesity Collaborators (2017). Health effects of overweight and obesity in 195 countries over 25 years. N. Engl. J. Med..

[2] Brown WA (2021). Single anastomosis duodenal-ileal bypass with sleeve gastrectomy/one anastomosis duodenal switch (SADI-S/OADS) IFSO position statement-update 2020. Obes. Surg..

[3] Enochs P (2020). Comparative analysis of the single-anastomosis duodenal-ileal bypass with sleeve gastrectomy (SADI-S) to established bariatric procedures: An assessment of 2-year postoperative data illustrating weight loss, type 2 diabetes, and nutritional status in a single US center. Surg. Obes. Relat. Dis..

[4] Sanchez-Pernaute A (2007). Proximal duodenal-ileal end-to-side bypass with sleeve gastrectomy: Proposed technique. Obes. Surg..

[5] Sánchez-Pernaute A (2022). Long-term results of single-anastomosis duodeno-ileal bypass with sleeve gastrectomy (SADI-S). Obes. Surg..

[6] Sánchez-Pernaute A (2015). Single-anastomosis duodenoileal bypass with sleeve gastrectomy (SADI-S) for obese diabetic patients. Surg. Obes. Relat. Dis..

[7] Topart P, Becouarn G (2017). The single anastomosis duodenal switch modifications: A review of the current literature on outcomes. Surg. Obes. Relat. Dis..

[8] Surve A (2020). Long-term outcomes of primary single-anastomosis duodeno-ileal bypass with sleeve gastrectomy (SADI-S). Surg. Obes. Relat. Dis..

[9] Salama AF (2023). Comparative analysis of 5-year efficacy and outcomes of single anastomosis procedures as revisional surgery for weight regain following sleeve gastrectomy. Surg. Endosc..

[10] Dindo D, Demartines N, Clavien PA (2004). Classification of surgical complications: A new proposal with evaluation in a cohort of 6336 patients and results of a survey. Ann. Surg..

[11] Schlegel A (2022). A multicentre outcome analysis to define global benchmarks for donation after circulatory death liver transplantation. J. Hepatol..

[12] Jones R (2009). Development of the GerdQ, a tool for the diagnosis and management of gastro-oesophageal reflux disease in primary care. Aliment. Pharmacol. Therap..

[13] Lee WJ (2013). Predicting success of metabolic surgery: Age, body mass index, C-peptide, and duration score. Surg. Obes. Relat. Dis..

[14] Rubino F (2017). Metabolic surgery in the treatment algorithm for type 2 diabetes: A joint statement by International Diabetes Organizations. Obes. Surg..

[15] Cottam A, Cottam D, Roslin M, Cottam S, Medlin W, Richards C, Surve A, Zaveri H (2016). A matched cohort analysis of sleeve gastrectomy with and without 300 cm loop duodenal switch with 18-month follow-up. Obes. Surg..

[16] Wang Z (2023). Based on propensity matching scores: Comparison of the efficacy of two kinds of bariatric surgery for obese type 2 diabetes. Obes. Surg..

[17] Gomberawalla A (2015). Predictors of success after laparoscopic sleeve gastrectomy. Bariatr. Surg. Pract. Patient Care.

[18] Lauti M, Kularatna M, Hill AG, MacCormick AD (2016). Weight regain following sleeve gastrectomy—A systematic review. Obes. Surg..

[19] Spinos D, Skarentzos K, Esagian SM, Seymour KA, Economopoulos KP (2021). The effectiveness of single-anastomosis duodenoileal bypass with sleeve gastrectomy/one anastomosis duodenal switch (SADI-S/OADS): An updated systematic review. Obes. Surg..

[20] Zaveri H (2018). Mid-term 4-year outcomes with single anastomosis duodenal-ileal bypass with sleeve gastrectomy surgery at a single US center. Obes. Surg..

[21] Balibrea JM (2017). Mid-term results and responsiveness predictors after two-step single-anastomosis duodeno-ileal bypass with sleeve gastrectomy. Obes. Surg..

[22] Cottam A (2016). A matched cohort analysis of sleeve gastrectomy with and without 300 cm loop duodenal switch with 18-month follow-up. Obes. Surg..

[23] Martini F (2016). Single-anastomosis pylorus-preserving bariatric procedures: Review of the literature. Obes. Surg..

[24] Shoar S, Poliakin L, Rubenstein R, Saber AA (2018). Single anastomosis duodeno-ileal switch (SADIS): A systematic review of efficacy and safety. Obes. Surg..

[25] Shen SC (2021). Efficacy of different procedures of metabolic surgery for type 2 diabetes in Asia: A Multinational and Multicenter Exploratory Study. Obes. Surg..

[26] Lee WJ (2015). Laparoscopic sleeve gastrectomy for type 2 diabetes mellitus: Predicting the success by ABCD score. Surg. Obes. Relat. Dis..

[27] Soong TC (2021). One anastomosis gastric bypass for the treatment of type 2 diabetes: Long-term results and recurrence. Obes. Surg..

[28] Surve A (2020). Early outcomes of primary SADI-S: An Australian experience. Obes. Surg..

[29] Cottam A (2018). An analysis of mid-term complications, weight loss, and type 2 diabetes resolution of stomach intestinal pylorus-sparing surgery (SIPS) versus Roux-En-Y Gastric Bypass (RYGB) with three-year follow-up. Obes. Surg..

[30] Wang Z (2023). Efficacy and safety of single-anastomosis duodenal-ileal bypass with sleeve gastrectomy for the treatment of Chinese T2D patients with obesity. Asian J. Surg..

[31] Lee MH (2020). Laparoscopic sleeve gastrectomy for type 2 diabetes mellitus: Long-term result and recurrence of diabetes. Obes. Surg..

[32] Cottam A (2017). A matched cohort analysis of stomach intestinal pylorus saving (SIPS) surgery versus biliopancreatic diversion with duodenal switch with two-year follow-up. Obes. Surg..

[33] Hickey MS (1998). A new paradigm for type 2 diabetes mellitus: Could it be a disease of the foregut?. Ann. Surg..

[34] Rodríguez A (2010). Small bowel obstruction after antecolic and antegastric laparoscopic Roux-en-Y gastric bypass: Could the incidence be reduced?. Obes. Surg..

[35] Scopinaro N (2006). Biliopancreatic diversion: Mechanisms of action and long-term results. Obes. Surg..

[36] Sánchez-Pernaute A (2015). Single-anastomosis duodenoileal bypass as a second step after sleeve gastrectomy. Surg. Obes. Relat. Dis..

[37] Sánchez-Pernaute A (2010). Single anastomosis duodeno-ileal bypass with sleeve gastrectomy (SADI-S). One to three-year follow-up. Obes. Surg..

[38] Sanchez-Pernaute A (2015). Single-anastomosis duodenoileal bypass with sleeve gastrectomy (SADI-S) for obese diabetic patients. Surg. Obes. Relat. Dis..

[39] Rao R, Mehta M, Sheth DR, Hogan G (2023). Four-year nutritional outcomes in single-anastomosis duodeno-ileal bypass with sleeve gastrectomy patients: An Australian experience. Obes. Surg..

[40] Pennestri F (2023). Robotic vs laparoscopic approach for single anastomosis duodenal-ileal bypass with sleeve gastrectomy: A propensity score matching analysis. Updates Surg..

[41] Vilallonga R (2015). Robotically assisted single anastomosis duodenoileal bypass after previous sleeve gastrectomy implementing high valuable technology for complex procedures. J. Obes..

[42] Wang L, Yu Y, Wang J, Li S, Jiang T (2022). Evaluation of the learning curve for robotic single-anastomosis duodenal-ileal bypass with sleeve gastrectomy. Front. Surg..

